# Evaluation of a digitally enhanced cardiac auscultation learning method in Cameroon: results of a controlled study

**DOI:** 10.1186/s12909-024-05501-3

**Published:** 2024-05-23

**Authors:** Georges Bediang, Agnès Baran à Zock, Fred-Cyrille Goethe Doualla, Chris Nganou-Gnindjio

**Affiliations:** Faculty of Medicine and Biomedical Sciences, University of Yaoundé I, P.O Box: 1364, Yaoundé, Cameroon

**Keywords:** Cardiac auscultation, Digitally enhanced learning, Medical students, Audio listening, Cameroon

## Abstract

**Background:**

Cardiac auscultation is an efficient and effective diagnostic tool, especially in low-income countries where access to modern diagnostic methods remains difficult. This study aimed to evaluate the effect of a digitally enhanced cardiac auscultation learning method on medical students’ performance and satisfaction.

**Methods:**

We conducted a double-arm parallel controlled trial, including newly admitted 4th -year medical students enrolled in two medical schools in Yaoundé, Cameroon and allocated into two groups: the intervention group (benefiting from theoretical lessons, clinical internship and the listening sessions of audio recordings of heart sounds) and the control group (benefiting from theoretical lessons and clinical internship). All the participants were subjected to a pretest before the beginning of the training, evaluating theoretical knowledge and recognition of cardiac sounds, and a post-test at the eighth week of clinical training associated with the evaluation of satisfaction. The endpoints were the progression of knowledge score, skills score, total (knowledge and skills) score and participant satisfaction.

**Results:**

Forty-nine participants (27 in the intervention group and 22 in the control group) completed the study. The knowledge progression (+ 26.7 versus + 7.5; p ˂0.01) and the total progression (+ 22.5 versus + 14.6; p ˂ 0.01) were higher in the intervention group with a statistically significant difference compared to the control group. There was no significant difference between the two groups regarding skills progression (+ 25 versus + 17.5; *p* = 0.27). Satisfaction was higher in general in the intervention group (p ˂ 0.01), which recommended this method compared to the control group.

**Conclusion:**

The learning method of cardiac auscultation reinforced by the listening sessions of audio recordings of heart sounds improves medical students’ performances (knowledge and global – knowledge and skills) who find it satisfactory and recommendable.

**Trial Registration:**

This trial has been registered the 29/11/2019 in the Pan African Clinical Trials Registry (http://www.pactr.org) under unique identification number PACTR202001504666847 and the protocol has been published in BMC Medical Education.

## Background

Auscultation is a technique that consists of listening to the sounds produced by the body’s organs using a stethoscope [1]. It is a diagnostic method that has undergone many developments since the time of Hippocrates, thanks to the many improvements made to the stethoscope [2–5]. The popularisation of the stethoscope has made general auscultation and cardiac auscultation an integral part of physical examination [6]. It is an accessible, non-invasive, effective and efficient diagnostic tool for screening cardiovascular diseases, particularly in countries with limited resources [2, 6, 7].

However, several studies demonstrate a decline in the knowledge and skills of medical students and practitioners in cardiac auscultation [6–9]. This decline is explained on the one hand by the democratisation of modern diagnostic tools (ECG, ultrasound), on the other hand, by the lack of development in methods of learning cardiac auscultation [6, 7].

The reference learning method of cardiac auscultation (known as the conventional method) has not evolved for almost 50 years. It combines a phase of theoretical courses and a practical phase at the patient’s bedside [6, 7]. The theoretical phase covers the cardiovascular system’s anatomical, physiological and semiological bases. During the practical phase, the teachers identify the heart sounds using a conventional stethoscope and describe them to the students, who must then recognise them in the patient. This method has many limitations, including the lack of integration between theoretical knowledge and practical skills, bedside teaching variability and limited patient access due to the high number of medical students [6, 10, 11].

Digital offers new perspectives in auscultation thanks to features such as processing (amplification and reduction of parasitic noise), recording and sharing of cardiac sounds. Thus, many alternative methods of auscultation learning based on digital technology have been tested to improve the knowledge and skills of practitioners. These include patient simulation (by software or mannequins), specialised learning software, mobile applications and electronic stethoscope [10, 12–16]. Simulation by a virtual patient and specialised software, is used to combine videos of real patients with the listening of cardiac recordings, while simulation by mannequins is used to enable learners to listen to synthetic heart sounds in conditions similar to the clinical environment [17–21]. Other alternative methods like mobile applications or multimedia support such as CDs are used to allow learners to have a variety of didactic content at hand [22–24]. Electronic stethoscopes or ultrasonic stethoscopes function like filtering, recording and storage of sounds, are used for instant or delayed listening of sounds by several learners [23].

A hybrid learning method is innovative, as it is based on the combination of the benefits of conventional learning methods and digital advances. Moreover, this approach is adapted to the context of resource-limited countries (characterised by an insufficient number of teachers and patients for a high number of students), where it could also improve the knowledge and skills of health professionals in auscultation as well as the quality of care offered [10, 12–16].

The general objective of this study was to evaluate the effect of a digitally enhanced cardiac auscultation learning method on medical students’ performance and satisfaction.

## Methods

More details on the methodology of this study have been published [25]. There were no significant changes to the initial protocol. The results of this trial are presented according to the guidelines of the CONSORT (Consolidated Standards of Reporting Trials) 2010 statement [26].

### Study design

This was a multicentre, double-arm, parallel (1:1) controlled trial in two centers in the city of Yaoundé. The intervention center was the Faculty of Medicine and Biomedical Sciences (FMBS), while the control center was the Higher Institute of Medical Technologies (HIMT). Participants meeting the inclusion criteria were allocated into two groups: the intervention group benefiting from theoretical courses, a clinical internship and listening sessions of audio recordings of heart sounds and the control group, benefiting from courses theory and a clinical internship. This study was conducted for six months, from September 1, 2021, to March 1, 2022. Participant monitoring and data collection were done throughout the study.

### Eligibility criteria

All medical students newly admitted to their 4th year of medical studies and consenting to participate were considered eligible. Repeaters were not included. Participants who had not fully taken part in (i) theoretical courses, (ii) clinical internships, (iii) 02 listening sessions (intervention group only), (iv) pretest and post-test (v), as well as those who withdrew their consent, were excluded.

### Informed consents

The informed consent of each participant was obtained during an interview, during which an information notice and a consent form were given and explained. No additional consent related to the collection and use of data or biological species was required.

### Intervention

#### Choice of comparator

The conventional method (theoretical courses and clinical training) was chosen as a comparator because it is the most widely used method for learning cardiac auscultation. This method combines lessons in cardiovascular semiology (3rd year of medical studies) with courses in cardiovascular pathology and clinical internships (4th year of medical studies).

#### Intervention group

Three activities were organised: theoretical courses, a clinical internship, and two audio listening sessions.

##### Theoretical courses

Three theoretical courses were prepared by the research team and validated by a committee of cardiologists. The goal of these courses was to remind people about some of the cardiovascular notions. The first course covered the anatomy and physiology of the cardiovascular system. The second was about cardiovascular semiology, and the third was about cardiac auscultation. These courses were done at the FMBS in 02 assignments: the first course was on the day of recruitment and the other two, three days after recruitment. Each course was presented on a computer, using PowerPoint software for one hour (30 min presentations and 30 min questions/answers). These courses were complementary to the clinical semiology courses delivered at the amphitheatre of the faculty during lectures or clinical internships in accordance with the 4th year of medical studies program.

##### Clinical internship

According to the official program of their medical school (internal medicine internship), participants in the intervention group underwent clinical training. These internships took place in four reference hospitals in Yaoundé (Yaoundé Hospital University Center, Yaoundé Central Hospital, Yaoundé General Hospital and Yaoundé Jamot Hospital). The internships began after the delivery of the theoretical courses for 8 weeks. The objectives were to introduce participants to the practice of physical examination (inspection, palpation, percussion, and auscultation), to reinforce the knowledge acquired during the learning of theoretical semiology and to improve their clinical observation writing skills. These objectives were achieved through the realisation of various activities such as clinical observation, follow-up of hospitalised patients, rounds and symposia. These activities were carried out under the supervision of department heads, residents and interns of different hospital departments.

##### Audio listening sessions

Five audios from actual patients recorded and stored in a database in 2017 in Cameroon were used [27]. These recordings were made using a Littmann© 3200 electronic stethoscope, annotated using Audacity© software and saved on a computer in WAV format. Each of the five recordings was one minute long and corresponded to a specific cardiac sound: B1 and B2 heart sounds, aortic stenosis murmur; B3 third heart sound; heart failure crackles and atrial fibrillation arrhythmia.

Two one-hour listening sessions for each of the audio files were carried out within the FMBS. Each session was organised in 3 parts: an introduction lasting 10 min, the actual listening of the five audio files lasting 40 min (04 series of 10 min) and feedback from the participants of a duration of 10 min. The audios were broadcast in a loop (series of 5 broadcasts) by speakers connected to a computer. Audacity software was used to read the audio files. During each series, each audio file was played for two minutes. The supervisor commented on the first series of each session to help participants to identify the different heart sounds. The broadcast recordings were given to the participants via a USB medium at the end of the second listening session for independent listening for 02 weeks. Text messages were sent to them to encourage to listen to these heart sounds (audio files).

#### Control group

##### Theoretical courses

The cardiac auscultation was learned within the control group using the classic method (theoretical courses and clinical internship). As in the intervention group, the participants took part in 03 theoretical courses within the HIMT, respectively addressing the anatomy and physiology of the cardiovascular system, cardiovascular semiology and cardiac auscultation.

##### Clinical internship

According to the official program of their medical school (internal medicine internship), participants in the intervention group underwent clinical training. These internships took place at the YHUC and the YCH after the theoretical course was delivered for eight weeks. The objectives and activities of the participants during this course were similar to those of the participants in the intervention group.

### Knowledge and skills assessments

Two evaluations (pretest and post-test) were organised for each group in their respective faculties. Each of these assessments had to evaluate the knowledge (theoretical part) and skills (practical part) of participants. The theoretical part was based on a multiple-choice questionnaire taken from the theoretical courses taught. The practical part was based on audio recognition of five heart sounds. The heart sounds (audio files) were broadcast using a loudspeaker connected to a computer. Audacity® software was used to defuse these sounds. After one minute of listening, participants had two minutes to answer a 3-item questionnaire for each sound. These items made it possible to assess two dimensions: the distinction between normal and abnormal sounds and the description of the sounds. At the end of evaluation of each participant, three scores (percentage) were available: the knowledge score, the skills score and the total score.

Pretest occurred at the study’s beginning, directly after the recruitment of participants Post-test happened at the end of the clinical internships, 60 days after the pretest. Regarding the post-test, the questions and sounds were similar to those of the pretest. Nevertheless, their order was changed by randomisation as described in the published protocol [25]. This modification was intended to remove the bias of memorising the order of the questions with the participants. Satisfaction was assessed during the post-test also.

### Judgment criteria

The baseline data were participants’ socio-demographic data, knowledge scores, skills scores and total pretest and post-test scores.

The primary endpoint was the progression of participants’ cardiac auscultation knowledge score in a group. It was based on the difference between the post-test knowledge score and the pretest knowledge score.

The secondary endpoints consisted of (i) progression of the participants’ cardiac auscultation skill score (difference between the post-test skill score and the pretest skill score), (ii) progression of the total score (difference between the total post-test score and the total pretest score) and (iii) participant satisfaction.

### Sample size

It was a probability and non-consecutive sampling. The starting hypothesis was that there was a difference in knowledge progression of 40% between the two groups. Indeed, in the pretest, the two groups will present 30% of correct answers. At the post-test, the percentage of correct answers will be 40% (control group) versus 80% (intervention group), i.e. a respective progression of 10% (control group) versus 50% (intervention group), a difference of 40% in knowledge progression between the two groups. For a margin of error (α = 5%) and statistical power (β = 80), the minimum sample size calculated on the Open Epi site was 46 participants (23 in the intervention group, 23 in the control group). Considering the risk of exclusion, the sample size was increased by 20%, i.e. 56 participants [28].

### Recruitment, allocation, randomisation, and principle of blinding

The participants were recruited and allocated directly from their training schools (those from the FMBS allocated to the intervention group and those from the HIMT allocated to the control group). The trial was non-randomised as the intervention group and the control group were defined according to the training schools of the participants. The participants’ group was unknown to the internship supervisors. The research team carried out all the interventions.

### Collection of data

The data was collected using 03 self-administered forms. The first two forms (pre and post-test) were multiple-choice questionnaires of 20 questions. The first 15 questions related to the knowledge from the theoretical courses, while the last 05 questions related to the recognition of the cardiac sounds described during the theoretical courses and the listening sessions. The final form focused on participant satisfaction. It consisted of 10 questions based on a 4-point Likert scale.

### Statistical analyses

Data from this study were presented as the mean (standard deviation) and median (interquartile range). The analyses consisted of a comparison between the two groups on the endpoints. The comparison tests used were the Student’s T-test for the quantitative variables following the normal distribution, the Mann-Whitney U test for the quantitative variables not following the normal distribution and the Chi-square for the categorical variables.

## Result

### Recruitment and flow of participants

**Fig. 1:**
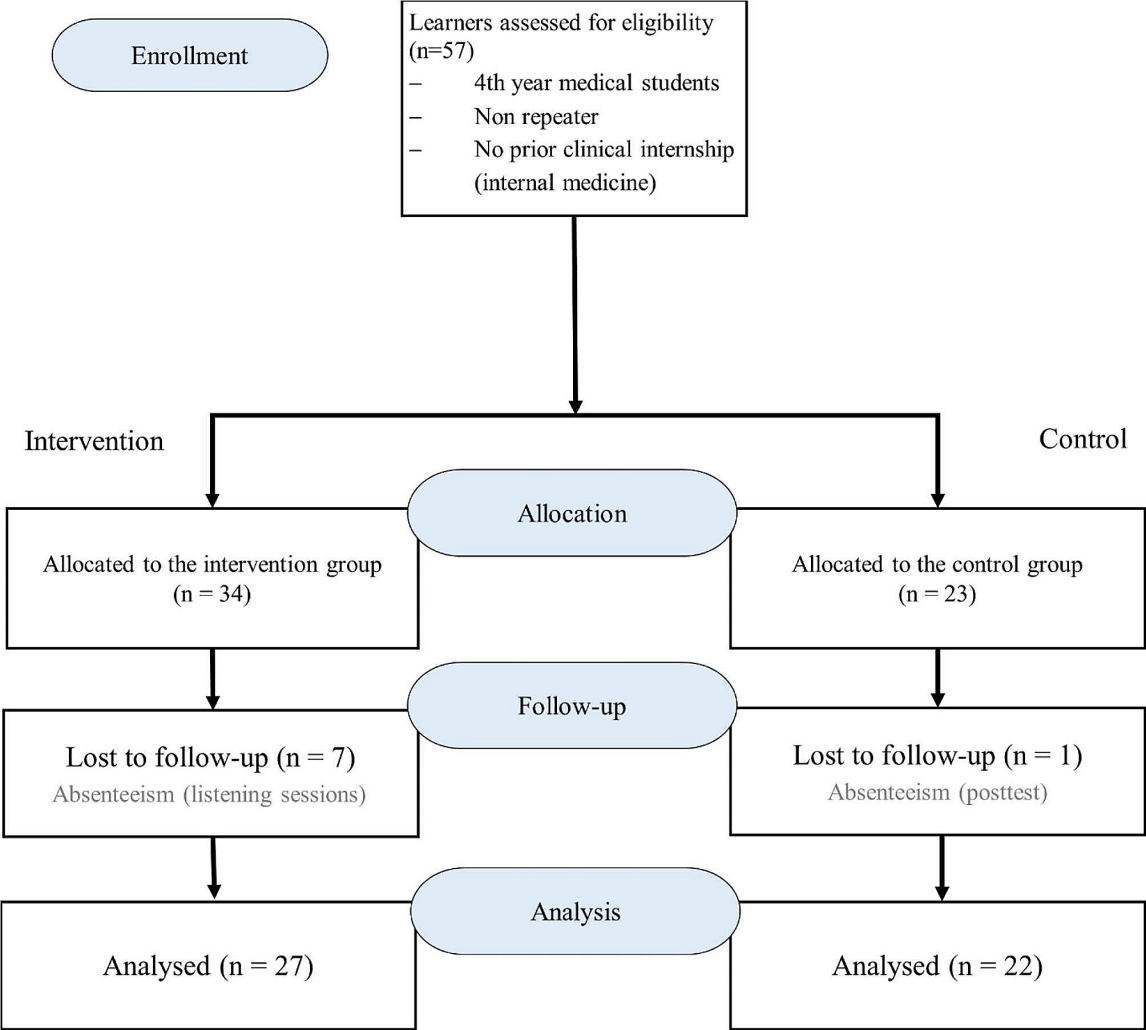
Participant flowchart

A total of 57 participants were included at the beginning of the study, with 34 participants allocated to the intervention group and 23 assigned to the control group. At the end of the study, the intervention group had 27 participants compared to 22 in the control group (Fig. 1).

### Participant characteristics

There was no statistically significant difference between the two groups in terms of gender (*p* = 0.41). The mean age in the intervention group was 21.6 ± 1.3 years, while 22.3 ± 2.1 years in the control group (*p* = 0.16) – Table 1.

**Table 1:** Participants’ characteristics

		Intervention group (*N* = 27)		Control group (*N* = 22)		*p*
Gender (female)		14		14		0.41
Internship sites						
	YUHC	6		6		
	YCH	8		16		
	YGH	6		0		
	YJH	7		0		
		**Mean ± SD**	**Min ; Max**	**Mean ± SD**	**Min ; Max**	
Age (years)		21.6 ± 1.3	19 ; 24	22.3 ± 2.1	20 ; 30	0.16

YUHC: Yaounde University Hospital Center, YCH: Yaounde Central Hospital, YGH: Yaounde General Hospital, YJH: Jamot Yaounde Hospital

### Pretest and post-test

At the post-test, a significant difference (*p* < 0.01) was observed between the 02 groups regarding the knowledge score. The average knowledge score in the intervention group was 73.3% against 64.9% in the control group. A significant difference (*p* < 0.01) was also observed between the total score of the intervention group (73.3%) and the total score of the control group (60.4%) as shown in Table 2.

### Primary outcome: knowledge score progression

For knowledge score progression, the intervention group had a percentage of + 26.7% versus + 7.5% in the control group with a statistically significant difference (*p* < 0.01) as described in Table 3.

**Table 2:** Participants’ scores in the pretest and post-test

		Intervention group (*N* = 27)		Control group (*N* = 22)		*p*	
		Med (IQR)	Min ; Max	Med (IQR)	Min ; Max		
Pretest scores (%)							
	Knowledge score	50 (40 ; 58)	18.3 ; 66.7	46.6 (35.8 ; 61.6)	13.3 ; 71.6	0.89	
	Skills score	50 (45 ; 65)	30 ; 85	50 (35 ; 56.3)	23.3 ; 95	0.18	
	Total (knowledge + skills) score	50.8 (45.8 ; 55)	31.7 ; 69.1	47.5 (40.6 ; 51.6)	30 ; 83.3	0.61	
Post-test scores (%)							
	Knowledge score	73.3 (66.7 ; 83.3)	46.6 ; 96.7	64.9 (35.8 ; 61.7)	18.3 ; 83.3	˂0.01	
	Skills score	80 (60 ; 90)	50 ; 95	60 (50 ; 75)	20 ; 90	0.05	
	Total (knowledge + skills) score	73.3 (66.7 ; 82.5)	55 ; 90.8	60.4 (52.7 ; 69.6)	19.2 ; 79.2	˂0.01	

IQR: interquartile range

**Table 3:** Primary and secondary outcomes

		Intervention group (*N* = 27)		Control group (*N* = 22)			*p*
		Med (IQR)	Min ; Max	Med (IQR)		Min ; Max	
Primary outcome							
	Knowledge progression score (%)	+ 26.7 (+ 13.3 ; +33.3)	+ 5 ; +71.7		+ 7.5 (-0.41 ; +22.1)	-8.34 ; 40.0	˂0.01
Secondary outcomes							
	Skills progression score (%)	+ 25 (+ 5 ; +40)	-15 ; +60		+ 17.5 (-10 ; +35)	-45 ; +55	0.27
	Total (knowledge + skills) progression score (%)	+ 22.5 (+ 13.3 ; +33.3)	-0.83 ; +48.3		+ 14.6 (+ 5.4 ; +22.3)	-26.7 ; 33.3	0.01

IQR: interquartile range

### Secondary outcomes

#### Skill score progression

Regarding the progression of the skill score, the intervention group had a percentage of + 25% against + 17.5% in the control group with no statistically significant difference (*p* = 0.27) as described in Table 3.

#### Total score progression

Regarding the progression of the total score, the intervention group had a percentage of + 22.5% against + 14.6% in the control group with a statistically significant difference (*p* = 0.01) - Table 3.

#### Participants satisfaction

Concerning participants’ satisfaction with learning methods, the intervention group had higher satisfaction than the control group on nine of the criteria studied (*p* < 0.01). All participants felt the need to receive additional learning: 4 (4 ; 4) versus 4 (4 ; 4) *p* = 0.86), as shown in Table 4.

**Table 4:** Secondary outcomes (satisfaction of participants)

		Intervention Group (*N* = 27)		Control Group (*N* = 22)		*P*
		Med (IQR)	Min ; Max	Med (IQR)	Min ; Max	
Easy application of the learning method		3 (2 ; 4)	2 ; 4	2 (2 ; 3)	1 ; 4	˂0.01
Definition of learning objectives		3 (3 ; 4)	3 ; 4	3 (2 ; 3)	1 ; 4	˂0.01
Theoretical courses were sufficiently developed		3 (3 ; 4)	2 ; 4	2 (2 ; 3)	1 ; 3	˂0.01
Clinical internships were sufficiently developed		3 (3 ; 3)	2 ; 4	2 (2 ; 3)	1 ; 4	˂0.01
Learning tools were appropriate		3 (3 ; 4)	2 ; 4	3 (2 ; 3)	1 ; 4	˂0.01
Learning tools were satisfactory		4 (3 ; 4)	2 ; 4	3 (2 ; 3)	1 ; 4	˂0.01
Defined learning objectives have been achieved		3 (3 ; 4)	2 ; 4	2.5 (2 ; 3)	1 ; 3	˂0.01
Learning methods were satisfactory		3 (3 ; 4)	2 ; 4	2.5 (2 ; 3)	1 ; 3	˂0.01
Ability of recommending this learning method		4 (3 ; 4)	3 ; 4	2.5 (2 ; 3)	1 ; 4	˂0,01
Need of additional learning		4 (4 ; 4)	3 ; 4	4 (4 ; 4)	1 ; 4	0.86

IQR: interquartile range

## Discussion

Cardiac auscultation is an efficient and effective diagnostic method for detecting many cardiovascular diseases, especially in countries with limited resources [29–31]. However, numerous studies highlight the decline in the performance of medical students and practitioners in cardiac auscultation [9, 15, 22, 32]. This decline is explained by the popularisation of modern diagnostic tools (echocardiography) and limited cardiac auscultation training. Many methods of learning cardiac auscultation have emerged to improve the performance of medical students and practitioners. The most common is based on the use of (i) patient simulation, (ii) specialised learning software, (iii) mobile applications and (iv) devices such as the electronic stethoscope [17–23, 33].

The learning method used in this study is based on the classic method of learning cardiac auscultation (theoretical courses and clinical internships) reinforced by audio listening sessions (supervised and unsupervised) of recordings of cardiac sounds aimed to reinforce the acquisition of knowledge and skills of participants (medical students).

### Knowledge and skills of pretest participants

In the pretest of this study, there was no statistically significant difference between the two groups regarding knowledge score (*p* = 0.89). These results differ from those of Stern et *al.* in 2001 in the USA, among students in their 3rd year of medical studies: the average score was 50.3% in the intervention group against 43.2% in the control group (p ˂ 0.05) [24]. This difference can be explained by the fact that in our study, the teaching program is the same in the two medical training schools (FMBS and HIMT) for equivalent levels of study. Without intervention, participants of similar levels received the same lessons, hence the identical knowledge scores.

Regarding the participants’ skills, there was no statistically significant difference between the two groups (*p* = 0.18). These results differ from those of Butter et *al.* in 2010 in the USA, who found an average of 67.3 ± 18.85% in the intervention group and 73.9 ± 14.1% in the control group (*p* = 0, 06) [14]. This difference is explained by the fact that in the study of Butter and *al.*, the comparison was made between students in the 3rd year of medical studies following learning by computer tutorial and patient simulator (intervention group, *n* = 77) and students in the 4th year of medical studies following theoretical courses and a clinical internship (control group *n* = 31). Fourth-year medical students have different lessons and more experience in auscultation and especially, during clinical internships. In our study, the teaching program and the level of study of the participants of the two groups are the same and all the participants included are naïve to the practice of auscultation.

### Knowledge and skills of post-test participants

About the post-test, there was a statistically significant difference between the two groups in terms of knowledge (*p* < 0.01). These results are similar to those of Stern and *al*. in 2001 in the USA, who found an average of 68.4 ± 11.6% in the intervention group against an average of 54.7 ± 13.8% in the control group (p ˂ 0.05) [24]. In our study, the improvement in knowledge can be explained by reinforcing knowledge acquired during classical learning through supervised listening sessions of heart sounds in the intervention group.

Regarding skills at the post-test, there was a statistically significant difference between the two groups regarding knowledge (*p* = 0.05). These results differ from those found by Mahnke et *al.* in 2004 in the USA, who observed an average of 51 ± 10% in the intervention group against 51.0 ± 14% in the control group with no statistically significant difference [15]. Several factors can explain these results. First, the Manhke et al. study participants were 1st and 2nd -year pediatric residents. In our study, the participants were 4th year medical students. Second, supervised learning time (listening sessions) was guaranteed and controlled in our study, unlike Manhke’s.

### Progression of knowledge and skills within groups

Regarding the progression of theoretical knowledge, there was a statistically significant difference between the two groups in favour of the intervention group (*p* < 0.01). These results are close to those of Stern and *al.* in 2001 in the USA, who found a progression of 18.2 ± 1.6% in the entire intervention group and 7.8 ± 0.6% in the control group (p ˂ 0.05) [24]. This progression could be attributed to additional supervised listening sessions associated with feedback.

Regarding the progression of skills, in our study, there was no statistically significant difference between the two groups (*p* = 0.27). These results are lower than those found by Barrett and *al.* in 2004 in the USA study whose the objective was to evaluate the effect of repetitive listening to audio files of cardiac sounds (250 to 500 repetitions in 6 sessions over one month) on cardiac auscultation skills in 2nd-year medical students [34].This study had observed a progression of 71.5 ± 7.8% in the complete intervention group versus 65.2 ± 4.7% in the group of partial intervention and 8 ± 1.1% in the control group with a statistically significant difference between the groups (p˂ 0.001). The lower progression in our study can be explained by: (i) the low frequency of supervised listening sessions in our study (02 sessions spread over 08 weeks) compared to the study by Barrett and *al.* in which 06 sessions of listening sessions were spread over 04 weeks, (ii) the time granted to the participants for independent listening (02 weeks in our study against 04 in that of Barrett and *al.*), (iii) and the impossibility of guaranteeing the effectiveness of independent listening sessions.

Finally, there was a statistically significant difference between the two groups in favour of the intervention group (*p* = 0.01) about the progression of knowledge and skills (total score). If the progression of skills was higher, we would have had an even higher progression of this total score.

### Limitations of the study

In this study, the main limitation was the inadequacy of the sample size compared to the initial forecasts. This mismatch is due to the Coronavirus pandemic, which has led to the cancellation of clinical internships and the temporary closure of academic institutions. Participants’ low voluntary participation rate also contributed to this small sample size.

## Conclusion

This study demonstrates that learning cardiac auscultation by a classic method reinforced by audio listening sessions significantly improves participants (students)’ knowledge and global (knowledge + skills) progression score. This method is satisfactory according to the participants who have benefited from it and would be an option for strengthening the method of learning cardiac auscultation in developing countries.

## Data Availability

All data generated and analysed during this study will be available from the corresponding author upon reasonable request.
